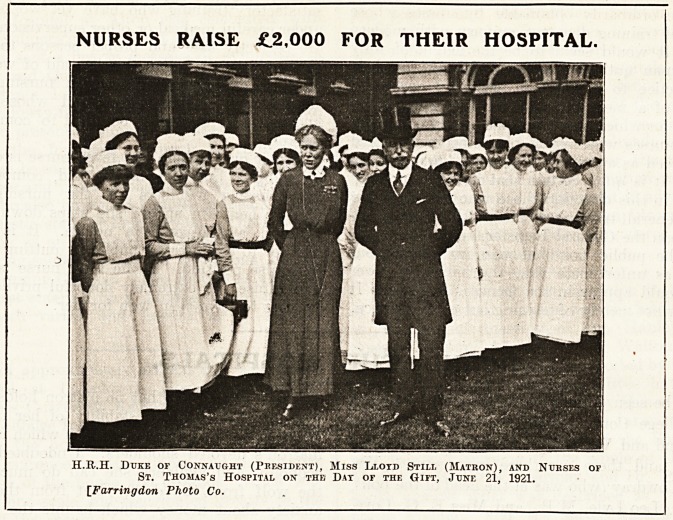# Fever Nurses and the General Register

**Published:** 1921-07-02

**Authors:** 


### THE MATRONS' AND SISTERS' DEPARTMENT.

### NURSES RAISE £2,000 FOR THEIR HOSPITAL.

H.R.H. Duke of Connaught (President), Miss Lloyd Still (Matron), and Nurses of St. Thomas's Hospital on the Day of the Gift, June 21, 1921.

[*Farringdon Photo Co.*

### FEVER NURSES AND THE GENERAL REGISTER.

The admirable course of training prescribed by the Scottish Board of Health for nurses in Scottish fever hospitals, crowned by an examination which is recognised by the State, has given rise to a curious situation under the Registration Act. The curriculum provides for a study of the principles of nursing, no less than for the study of anatomy and physiology, hygiene, and dietetics. The Scottish Board of Health has accordingly put in a claim on behalf of all those nurses who have taken their fever nurses' certificate that they should be admitted during the period of grace to the General Nurses' Register, as well as (if they please) to the Special Fever Nurses' Register. This claim, in spite of the opposition offered by the General Nursing Council for Scotland, appears to have been sustained, though negotiations are still on foot with a view to a settlement. As the draft rules stand, the General Nursing Council for Scotland must admit these fever nurses to the General Register. And since there is compulsory reciprocity, whether they will or no, the General Nursing Council for England and Wales must admit them likewise should they desire to place their names on the General Register.

Dr. Ker, in the interesting and lucid letter which we published last week, makes out a strong case, it seems to us, against the existence of any supplementary register at all during the period of grace. Few will deny that three years of special training in a well-equipped fever hospital is better than no training at all, and possibly better than a year's course in a minor Poor-Law infirmary or other small institution. Therefore, it is contended, if a place on the General Register be denied to certificated fever nurses they are set lower than the lowest type of *bona-fide* nurses whose claim to admission can be allowed. The very energy and success they have shown and the qualities of which they have given evidence in the course of their training are positively brought up against them in assigning them their status as nurses in the public eye. The same argument could be used for the admission of mental or children's nurses to the General Register. From this point of view the Scottish Board of Health is justified in its action. Indeed, the presence of the Scottish fever nurses on the General Register will probably have to be accepted as one of the many temporary anomalies produced by such co-ordinating measures as the Registration Acts.

It by no means follows that it is a good thing either for the nurses themselves or the public that the names of fever nurses should be placed on the General Register. From the point of view of the nurses it reduces them from the status of highly-trained specialists, entitled to registration by training and certification, to whom a lucrative branch of the profession is open as by right, to the status of *bona-fide* nurses admitted by grace for whom apologies have to be made. It is a little ridiculous to regard fever nursing as an inferior branch of nursing or the Fever Register as less honourable than the General Register. The fees earned by private fever nurses are from 25 to 50 per cent. higher than those ordinarily obtainable by nurses whose standard of training does not conform with modern notions. It would seem to be the height of folly for a woman entitled to participate in the best fever practice to descend of her own accord to the level of a mere *bona-fide* in a branch of the profession for which she is not adequately equipped. All fever nurses who aim at obtaining institutional posts proceed as a matter of course to take general training. It is quite certain that it will in no way advantage in this direction those who do not desire to take general training to have their names inscribed upon the General Register.

From the public point of view we cannot but regard it as unfortunate that the names of fever nurses should appear in the General Register. It is a matter not merely of training but of experience. There may be many *bona-fide* nurses with very fragmentary and (from the modern aspect) unsatisfactory training who have yet worked so long under careful medical or other supervision that they are perfectly safe and proper persons to have the care of patients. Can this be said of women who have no experience whatever in nursing ordinary surgical and medical cases and whose practical knowledge of disease is confined to communicable disorders?

In her own sphere the fever nurse is one of the most highly qualified, highly paid, competent, and indispensable members of the nursing service. Outside her own sphere she steps down from her pedestal and becomes a novice. It is a radical blunder to confuse the public by putting her name in a false position. If the fever nurse be wise she will refuse the extremely doubtful privilege which appears to have been won for her.

## Figures and Tables

**Figure f1:**